# Baicalein Exerts Therapeutic Effects against Endotoxin-Induced Depression-like Behavior in Mice by Decreasing Inflammatory Cytokines and Increasing Brain-Derived Neurotrophic Factor Levels

**DOI:** 10.3390/antiox11050947

**Published:** 2022-05-11

**Authors:** Hsin-Tzu Liu, Yu-Ning Lin, Ming-Cheng Tsai, Ya-Chi Wu, Ming-Chung Lee

**Affiliations:** 1 Department of Medical Research, Hualien Tzu Chi Hospital, Buddhist Tzu Chi Medical Foundation, Hualien 970, Taiwan; htl1@ms43.hinet.net (H.-T.L.); a100711012@gmail.com (Y.-C.W.); 2 Department of Chinese Medicine, Hualien Tzu Chi Hospital, Buddhist Tzu Chi Medical Foundation, Hualien 970, Taiwan; yuning.lin@msa.hinet.net; 3 Department of Neurosurgery, Shin-Kong Wu Ho-Su Memorial Hospital, Taipei 111, Taiwan; m000679@ms.skh.org.tw; 4 Department of Life Science, National Taiwan Normal University, Taipei 116, Taiwan; 5 School of Life Science, National Taiwan Normal University, No. 88, Sec. 4, Ting-Zhou Road, Taipei 116, Taiwan

**Keywords:** baicalein, depression-like behavior, pro-inflammatory cytokines, brain-derived neurotrophic factor

## Abstract

Inflammation plays an important role in the pathophysiology of depression. This study aims to elucidate the antidepressant effect of baicalein, an anti-inflammatory component of a traditional Chinese herbal medicine (*Scutellaria baicalensis*), on lipopolysaccharide (LPS)-induced depression-like behavior in mice, and to investigate the underlying mechanisms. In vitro, baicalein exhibited antioxidant activity and protected macrophages from LPS-induced damage. The results of the tail suspension test and forced swimming test (tests for despair potential in mice) showed the antidepressant effect of baicalein on LPS-treated mice. It also substantially decreased the production of pro-inflammatory cytokines, including IL-6, TNF-α, MCP-1, and eotaxin, elicited by LPS in the plasma. Baicalein downregulated NF-κB-p65 and iNOS protein levels in the hippocampus, demonstrated its ability to mitigate neuroinflammation. Additionally, baicalein increased the levels of the mature brain-derived neurotrophic factor (mBDNF) in the hippocampus of LPS-treated mice, and elevated the ratio of mBDNF/proBDNF, which regulates neuronal survival and synaptic plasticity. Baicalein also promoted the expression of CREB, which plays a role in a variety of signaling pathways. In summary, the findings of this study demonstrate that the administration of baicalein can attenuate LPS-induced depression-like behavior by suppressing neuroinflammation and inflammation induced by the peripheral immune response.

## 1. Introduction

Depression is a common mental illness in which an individual persistently experiences sadness and loss of pleasure for long durations, and has difficulty participating in normal routines. In modern society, lifestyles are associated with chronic stress, which commonly leads to the dysregulation of the Hypothalamic-Pituitary-Adrenal (HPA) axis and elevated glucocorticoid hormone levels. Previously, the occurrence of depression and neuronal damage have been observed in rodents with chronic exposure to glucocorticoids [1,2]. According to the 2017 World Health Organization (WHO) report, 322 million people are estimated to suffer from depression, which has become the leading cause of disability, and contributes to disease burden globally [3].

The monoamine deficiency theory is the most well-established etiological mechanism of depression. Depletion of monoamine-series neurotransmitters, such as serotonin (5-HT) and dopamine, contributes to depression development. Monoamine levels can increase rapidly after the administration of an antidepressant, but the potential beneficial effects of the treatment are delayed by weeks, indicating that other underlying mechanisms are involved [4,5,6].

Evidence accumulated in the last two decades supports the vital role of pro-inflammatory cytokines in the etiology and pathogenesis of depression [7]. Meta-analytic research has shown that patients with major depressive disorder (MDD) have significantly higher serum IL-6 and TNF-α levels than healthy controls [8]. Both antidepressants and successful psychotherapy can lead to a decrease in pro-inflammatory cytokine levels. The concordance of elevated pro-inflammatory cytokine levels between serum and brain was detected after the administration of lipopolysaccharide (LPS) in mice [9], suggesting that peripheral inflammation can activate neuroinflammation (the inflammatory response of the brain). Neuroinflammation influences depression and neurodegeneration, an effect attributed to the dysregulation of cytokines [10,11]. Additionally, it has been hypothesized that pro-inflammatory cytokines can induce a deficit in dopamine and serotonin production [12].

The brain-derived neurotrophic factor (BDNF) has been implicated in depression [13,14]. The involvement of BDNF has been demonstrated in the progression of several brain diseases, including Parkinson’s and Alzheimer’s diseases; patients with such diseases typically present with the comorbidity of depressive disorder [15,16]. Many clinical studies conducted among patients with MDD have shown elevated serum BDNF levels after the administration of effective antidepressant treatment [17]. Studies with animal models have demonstrated that stress could reduce BDNF expression in the brain, whereas antidepressant treatment could restore it [18]. Moreover, the reduction of the BDNF levels is proposed to be associated with neuroinflammation, and it could lead to a reduction in hippocampal neurogenesis [19,20]. In this study, the BDNF was regarded as a biomarker of depression.

There is reason to believe that anti-inflammatory treatment may be useful for depressive disorders. The root extract of *Scutellaria baicalensis Georgi*, a traditional Chinese herbal medicine that is widely used to treat inflammation, exhibits anti-inflammatory activity, including inhibition of pro-inflammatory cytokine production [21]. It has been demonstrated that treatment with the extract could normalize the depression-like behavior of stressed rats [22]. Baicalin, one of the compounds in the extract, has been previously reported for its anti-inflammatory and antidepressant effect on rodent models [23,24,25,26,27]. Baicalein, the aglycone of baicalin, is also a main bioactive component of the extract [28] and is proven to inhibit pro-inflammatory cytokine production in LPS-induced mastitis mice [29]; however, its therapeutic effect on LPS-induced depression is still unclear. As a gram-positive bacterial endotoxin, LPS can activate the production of pro-inflammatory cytokines, and depression-like behaviors can be observed 24 h post peripheral LPS administration in mice. LPS-induced depression is a specific rodent model to explore inflammation-associated behavioral responses [30,31]. Although mice modeled with chronic unpredictable mild stimulation (CUMS) are more commonly used to explore depression, the associated mechanisms of the behavioral responses are more complicated [26].

This study aims to elucidate whether baicalein could exert antidepressant effects by attenuating inflammation and increasing BDNF expression, thereby mitigating depression in mice. The LPS-induced model was used for this investigation. Moreover, we also aim to explore the possible signaling pathways involved in mediating the antidepressant effect of baicalein. 

## 2. Materials and Methods

### 2.1. Animals and Drugs

The experiments were performed with 8-week-old male C57BL/6 mice (weight: 20–30 g) obtained from the National Laboratory Animal Center. Four animals were housed per cage at room temperature (25 °C) with 12 h light and dark cycles and ad libitum access to food and water. Animal use was approved by the Institutional Animal Care and Use Committee (IACUC) of Tzu-Chi Hospital (approval number 107-18). The total number of mice used in this study was about 45.

All chemicals used in this study were of analytical grade. Lipopolysaccharide (L2880, *Escherichia coli* 055:B5) and baicalein were purchased from Sigma-Aldrich Co. (St. Louis, MO, USA). Both reagents were freshly prepared prior to each experiment.

### 2.2. Experimental Design and Drug Treatments

Mice were randomly assigned into three groups as follows (*n* = 6–8 mice per group):

1. Group I was treated with normal saline twice with a gap of 1 h between treatments. This group was considered the normal control group.
2. Group II was injected with LPS (5 mg/kg, i.p.) and treated with normal saline after 1 h (LPS-treated mice).
3. Group III was injected with LPS (5 mg/kg, i.p.) followed by a one-time administration of baicalein (3 mg/kg, i.p.) after 1 h (LPS-Baicalein-treated mice).

A control group treated with baicalein, which may show the effect of baicalein on healthy animals, was not included because the goal of this study was to evaluate the therapeutic effect of this compound. The determined doses of baicalein (3 mg/kg, i.p.) and LPS (5 mg/kg, i.p.) were based on previous studies [29,32,33]. 

In order to know if baicalein could inhibit inflammation when LPS commenced its effects, baicalein was injected 1 h after LPS administration. Behavioral evaluations were performed 24–27 h after LPS administration (23–26 h after baicalein administration). Then, the mice were sacrificed the same day that the final behavioral test was performed. The time between the baicalein administration and sacrifice was about 26 h; this was almost the same for all animals.

### 2.3. Behavioral Evaluations

As the depression-like behaviors can be observed 24 h post peripheral LPS administration in mice [34,35], behavioral tests were conducted with animals in all groups 24–27 h after LPS administration. The forced swimming test (FST) and tail suspension test (TST) are widely used to assess depression-like behavior in rodent models [36,37]. The duration of immobility was measured to evaluate the potential for behavioral despair. 

In this study, different batches of mice were used for TST and FST. Each mouse underwent either FST or TST. Half of the total mice were randomly assigned into three groups for different treatments; then, the mice underwent TST on the same day. Moreover, the other half underwent FST; however, TST and FST were not conducted on the same day. Each test was performed at almost the same time after baicalein administration.

#### 2.3.1. Forced Swimming Test (FST)

Mice were individually placed in an acrylic cylinder (height: 40 cm, diameter: 18 cm) filled with 30 cm of water (25 ± 1 °C) to prevent them from touching the bottom. The activity of each mouse was videotaped during a 6 min swimming session. Following the session, the mice were towel-dried and placed under a 60 W bulb for further drying. The water in the acrylic cylinder was changed after each trial. Immobility was defined as the mice floating in the water without struggling and only making gentle movements to keep their head above the water. The total immobility time was measured during the 5 min of the swimming session. The FST recordings were analyzed using the commercial video tracking software SMART 3.0 (Panlab Harvard Apparatus, Barcelona, Spain).

#### 2.3.2. Tail Suspension Test (TST)

The mice were individually suspended 30 cm above the floor of the suspension box. The tape securely adhered the tail of the mouse to the suspension bar. Each mouse was suspended in the middle of the compartment. The contact with the walls was disabled, and the activity of the mice was videotaped during a 6 min session. At the end of each session, the mouse was returned to the home cage, and the apparatus was wiped thoroughly. Immobility was defined as the observation of a completely motionless, suspended animal. The total immobility time was measured during the 5 min of the session. TST analysis was also performed using SMART 3.0.

### 2.4. Cytokine Assessment

After the final behavioral test, the mice were anesthetized with 3% isoflurane (Panion & BF Biotech, Taoyuan, Taiwan), and blood samples were collected from the hearts. The blood samples were centrifuged at 3000 rpm for 10 min at 4 °C to obtain the plasma. Concentrations of circulating cytokines, including IL-6, IL-1β, IL-10, eotaxin, monocyte chemoattractant protein-1 (MCP-1), and TNF-α, were assessed using the MILLIPLEX Multi-Analyte Profiling (MAP) Mouse Cytokine/Chemokine Kit (Millipore, Billerica, MA, USA) on a Luminex platform.

### 2.5. Assessment of in Vivo Anti-Oxidation Ability

Mouse plasma of all groups was used to detect the antioxidant activities against hydrogen peroxide using a chemiluminescence (CL) analysis system. The CL signal emitted from the mixture containing 200 µL of plasma and 500 µL of luminol was measured with an ultrasensitive CL analyzer (CLD-110; Tohoku Electronic Industrial, Japan) as the baseline level. Then, 100 µL of 0.03% H_2_O_2_ (Nihon Shiyaku Reagent, Tokyo, Japan) was loaded into the mixture for continuous signal recording. Total CL counts in 5 min were calculated by integrating the area under the curve. The results are expressed as CL counts (10 s)^−1^. The assay was carried out in duplicate for each sample.

### 2.6. Western Blot Analysis

The mice were anesthetized and euthanized by cervical dislocation after the final behavioral test. The mouse hippocampi were dissected and homogenized in RIPA buffer containing protease and phosphatase inhibitors. Tissue lysates were centrifuged for 10 min at 4 °C, and the supernatants were collected for the protein concentration assay, which was conducted using a BCA kit (Bio-Rad Laboratories, Hercules, CA, USA). SDS-PAGE electrophoresis was used to separate proteins, and the proteins were subsequently transferred onto a PVDF membrane. Subsequently, the membranes were incubated in solutions containing primary antibodies at 4 °C overnight. The primary antibodies used in this study were anti-iNOS (GTX130246, GeneTex, Irvine, CA, USA), anti-BDNF (ab226843, Abcam, Cambridge, MA, USA), anti-NF-κB p65 (8242S, Cell Signaling Technology, Beverly, MA, USA), anti-CREB (9197S, Cell Signaling Technology, Beverly, MA, USA), and GAPDH (sc-32233 Santa Cruz Biotechnology, Santa Cruz, CA, USA). After washing, the membranes were incubated with a horseradish peroxidase-conjugated (HRP) secondary antibody (Santa Cruz Biotechnology, Santa Cruz, CA, USA) for 1 h at room temperature. Finally, a chemiluminescent HRP substrate was used to visualize the protein bands. 

### 2.7. Macrophage Viability Assay

Using the 3-(4,5-dimethylthiazol-2-yl)-2,5-diphenyltetrazolium bromide (MTT) assay, which detects viable mitochondria in active cells, we examined the effect of baicalein on the viability of liver macrophages (Kupffer cells) (SCC119, Sigma-Aldrich Co., Saint Louis, Missouri, USA). Briefly, baicalein (1–100 µM) mixed with LPS was added to the medium, and the cells were incubated at 37 °C for 24 h. Then, MTT reagents were added to the medium and incubated for another 2 h. After discarding the medium, the cells were collected, and DMSO was added to dissolve the MTT formazan. The end color of the assay was analyzed by measuring the absorbance at 570 nm, which was proportional to the cell number. The assay was carried out in triplicate for each sample.

### 2.8. DPPH Radical Scavenging Assay

The free radical scavenging activity of baicalein was determined in vitro using the DPPH assay. Various concentrations of baicalein (5–1000 µM) were mixed with a methanol solution of DPPH and shaken vigorously. The mixture was then left in the dark for 30 min. The absorbance was measured at 517 nm, and the DPPH radical scavenging efficiency was calculated as follows:

DPPH radical scavenging efficiency (%) = (1 − absorbance of sample/absorbance of control) × 100(1)

L-ascorbic acid was used as the standard. The experiment was performed in triplicate for each sample.

### 2.9. Statistical Analysis 

Statistical analyses were performed using GraphPad Prism 6.0 (GraphPad Software Inc., San Diego, CA, USA). Data are presented as the mean ± SEM. Comparisons between two groups were performed using the Mann–Whitney U-test, and the results of the DPPH assay were analyzed using one-way ANOVA and Tukey’s test. The Shapiro–Wilks test was adopted to detect the departure from normality. Levene’s test was used to assess the homogeneity of variance across samples. Statistical significance was set at *p* < 0.05. 

## 3. Results

### 3.1. Baicalein Exhibits Antioxidant Activity in a Dose-Dependent Manner

**Figure 1:**
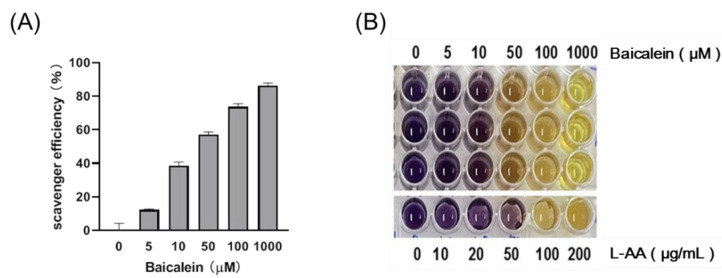
The free radical scavenging ability of baicalein, as assessed using the DPPH assay. (**A**) Baicalein scavenges free radicals in a dose-dependent manner. (**B**) The results of the DPPH assay.

The 2,2-diphenyl-1-picryl-hydrazyl-hydrate (DPPH) assay was performed in vitro to evaluate the antioxidant activities of baicalein. The higher the concentration of baicalein, the higher the antioxidant activity (Figure 1).

### 3.2. Baicalein Effectively Relieves LPS-Induced Damage in Liver Macrophages

**Figure 2:**
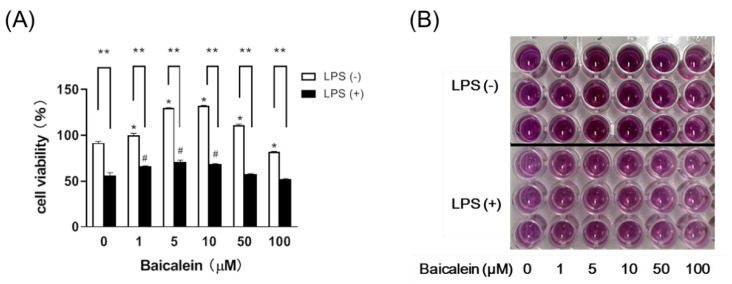
Baicalein effectively relieves LPS-induced damage in liver macrophages, as assessed by the MTT assay. (**A**) The viability of liver macrophages increased upon treatment with 1–10 µM of baicalein. Data are presented as means ± SEM. * *p* < 0.05 and # *p* < 0.05 indicate significant differences compared with the respective control group (0 µM Baicalein). Administration of LPS (+LPS) in the cell medium markedly reduced viability, but treatment with baicalein significantly restored the viability of macrophages. Data are presented as means ± SEM. ** *p* < 0.01 indicates significant differences compared with the respective LPS (−) group. (**B**) The results of the MTT assay.

According to our results, baicalein increased the viability of the macrophages alone and demonstrated effects against LPS at concentrations ranging from 1–10 µM (Figure 2).

### 3.3. Baicalein Treatment Mitigates LPS-Induced Depression-like Behavior

**Figure 3:**
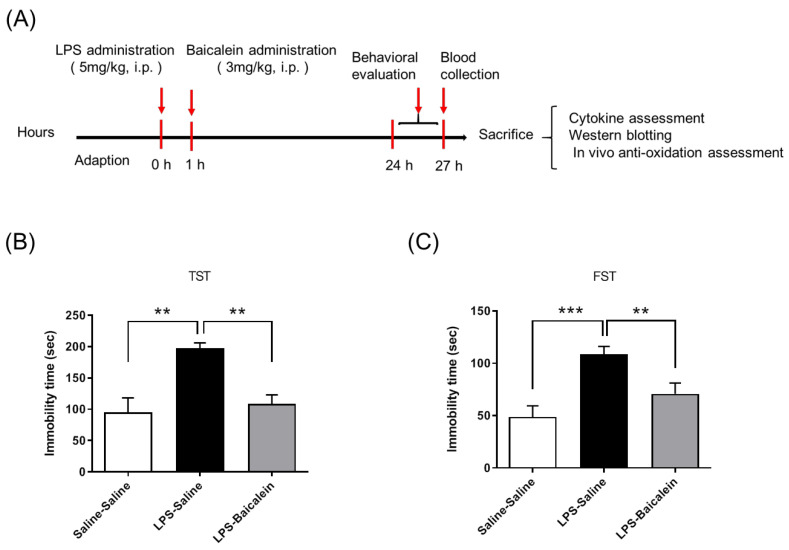
Study design and therapeutic effects of baicalein in mitigating LPS-induced depression-like behaviors. The time schedule of the study design (**A**). The control group was administrated with normal saline twice (at 0 h and 1 h). The LPS-treated group was administrated with saline instead of baicalein at 1 h. LPS-induced depression-like behavior was evaluated using the tail suspension test (**B**) and forced swimming test (**C**). Both tests show that immobility duration increased after administration of LPS (5 mg/kg i.p.). Treatment with baicalein (3 mg/kg i.p.) 1 h after LPS administration markedly reduced the immobility duration and improved depression-like behaviors. Data are presented as means ± SEM. (*n* = 6) ** *p* < 0.01, *** *p* < 0.001.

Both behavioral tests, FST and TST, showed that the duration of immobility increased after intraperitoneal (i.p.) administration of LPS (5 mg/kg), compared with that observed in the control group. Treatment with baicalein (3 mg/kg, i.p.) 1 h after LPS administration significantly decreased the immobility duration, indicating that it could normalize depression-like behaviors induced by LPS (Figure 3).

### 3.4. Bacalien Mitigates the Increase in the Concentrations of Circulating Cytokines Induced by LPS

**Figure 4:**
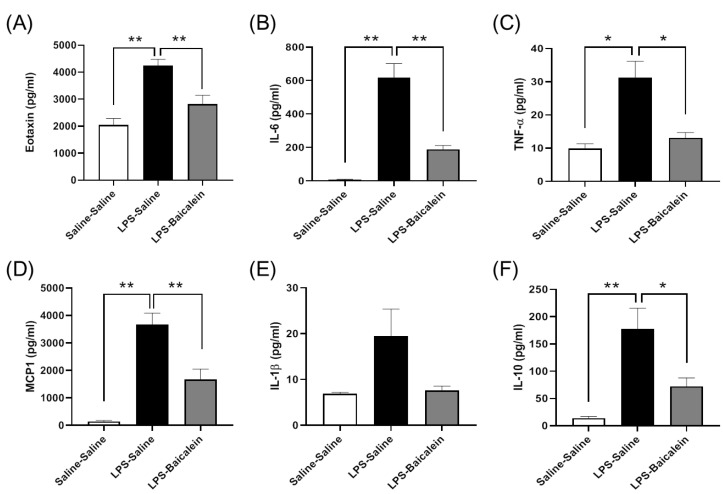
Effect of baicalein on the levels of cytokines in the plasma of mice. (**A**–**F**) The levels of cytokines increased in plasma after the administration of LPS (5 mg/kg, i.p.). Treatment with baicalein (3 mg/kg, i.p.) after 1 h following LPS administration significantly reduced the levels of cytokines. (**A**) Eotaxin. (**B**) IL-6: interleukin-6. (**C**) TNF-α: tumor necrosis factor-α. (**D**) MCP1: monocyte chemoattractant protein-1. (**E**) IL-1β: interleukin 1β. (**F**) IL-10: interleukin-10. Data are presented as means ± SEM. (*n* = 6) * *p* < 0.05, ** *p* < 0.01.

The concentrations of circulating cytokines, except those of IL-1β, increased significantly after the systemic challenge with LPS. The levels of IL-6, IL-10, eotaxin, monocyte chemoattractant protein-1 (MCP-1), and TNF-α were significantly downregulated in mice treated with both LPS and baicalein. The results showed that baicalein effectively moderated the peripheral immune response and inhibited inflammation (Figure 4).

### 3.5. Baicalein Improves the Free Radical Scavenging Activity in Plasma

**Figure 5:**
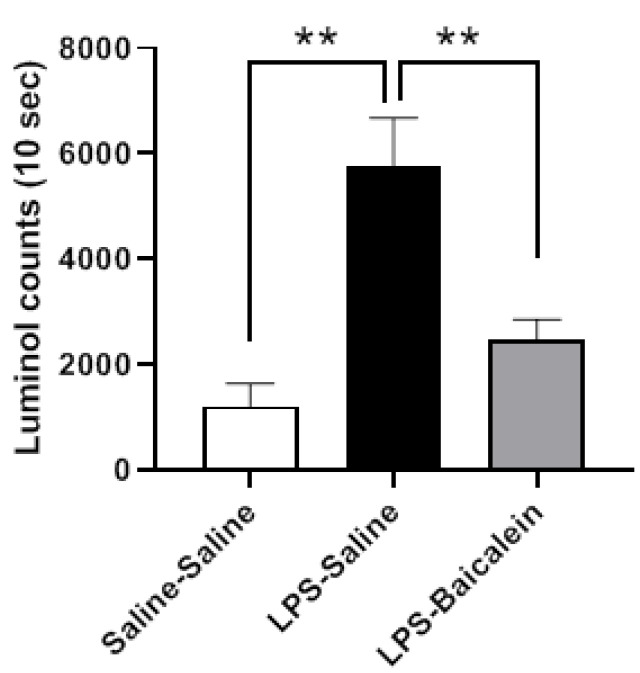
Effect of baicalein on the H_2_O_2_- scavenging activity in vivo. The H_2_O_2_- scavenging activity decreased after administration of LPS (1 mg/kg, i.p.), as assessed using a luminol-amplified chemiluminescence test. Treatment with baicalein (3 mg/kg, i.p.) 1 h after LPS administration markedly increased the H_2_O_2_-scavenging activity. Data are presented as means ± SEM. (*n* = 6) ** *p* < 0.01.

Compared with the control group, the plasma of LPS-treated mice exhibited reduced scavenging activity for H_2_O_2_, which was restored after baicalein administration. Baicalein clearly demonstrated beneficial effects against LPS-induced oxidative stress under in vivo conditions (Figure 5).

### 3.6. Treatment with Baicalein Significantly Reduces NF-κB-p65 and iNOS Protein Levels Elicited by LPS in the Hippocampus

**Figure 6:**
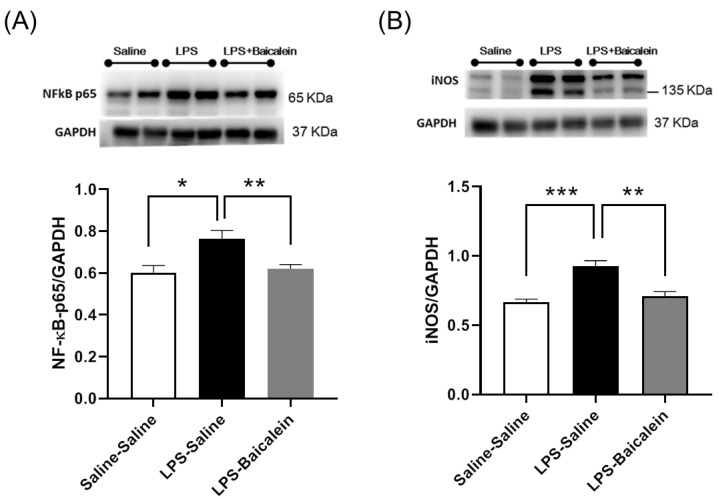
Effect of Baicalein on the protein levels of NF-κB-p65 and iNOS in the hippocampi of mice. Levels of NF-κB-p65 (**A**) and iNOS (**B**) in the hippocampus increased after administration of LPS (5 mg/kg, i.p.). Treatment with baicalein (3 mg/kg, i.p.) 1 h after LPS administration could significantly reduce the levels of both proteins. Data are presented as means ± SEM. (*n* = 8) * *p* < 0.05, ** *p* < 0.01, *** *p* < 0.001.

To evaluate inflammation in the hippocampus, NF-κB-p65 and iNOS protein levels were measured via western blotting. As iNOS is the downstream target of NF-κB-p65 during the induction of the inflammatory response, both NF-κB-p65 and iNOS levels increased significantly in LPS-treated mice, but decreased significantly in the LPS-baicalein-treated mice (Figure 6); hence, baicalein was shown to reduce neuroinflammation.

### 3.7. Baicalein Promotes the Protein Expression of BDNF and CREB Inhibited by LPS in the Hippocampus

**Figure 7:**
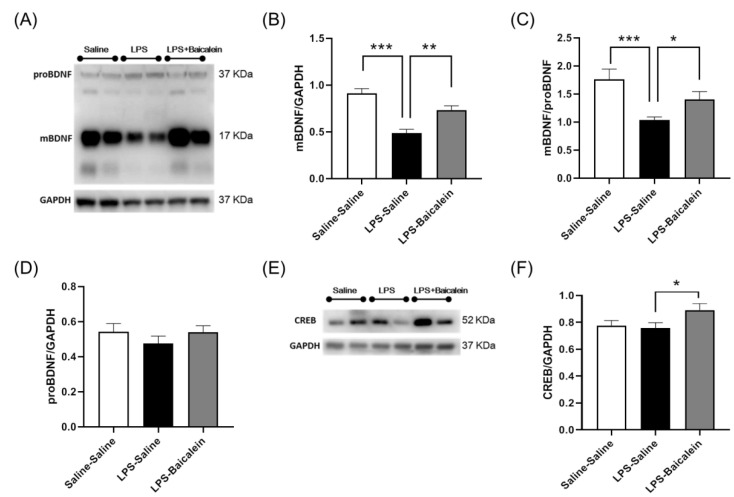
Effect of baicalein on the protein levels of BDNF and CREB in the hippocampi of mice. (**A**) Western blots showing levels of mBDNF and proBDNF. The levels of mBDNF (**B**) and the ratio of mBDNF/proBDNF (**C**) in the hippocampus decreased significantly after the administration of LPS (5 mg/kg, i.p.). Treatment with baicalein (3 mg/kg, i.p.) 1 h following LPS administration could inhibit the reduction. The levels of proBDNF (**D**) did not differ among the groups. (**E**) Western blots showing levels of CREB. Baicalein promoted a significant increase in the expression of CREB protein (**F**), as observed in LPS-Baicalein-treated mice. Data are presented as means ± SEM. (*n* = 8) * *p* < 0.05, ** *p* < 0.01, *** *p* < 0.001.

To elucidate the antidepressant mechanism of baicalein, we examined the protein levels of BDNF and cAMP-response element-binding protein (CREB) in the hippocampus. The results showed that LPS inhibited the production of mBDNF (Figure 7A,B) but not that of proBDNF. Baicalein treatment restored the level of mBDNF, and as a result, the ratio of mBDNF/proBDNF increased significantly (Figure 7A–D). Compared with the control group, CREB levels changed slightly in LPS-treated mice. Interestingly, baicalein significantly promoted CREB protein expression, as observed in LPS-baicalein-treated mice (Figure 7E–F).

## 4. Discussion

Anti-inflammatory treatments have been proposed as a therapeutic approach for depression [38]. The anti-inflammatory effects of baicalein have been demonstrated in conjunction with inflammation-related diseases [39]. Baicalein could suppress the release of pro-inflammatory mediators, including TNF-α, IL-6, and iNOS, in RAW264.7 macrophages stimulated by LPS [40]. Based on these findings, we hypothesized that baicalein might attenuate inflammatory responses and depression-like behaviors in mice. The results of this study confirmed our hypothesis. Baicalein exerted both anti-inflammatory and antidepressant effects.

An increase in the levels of circulating pro-inflammatory cytokines synchronized with depressive behavior was observed in LPS-treated mice, as expected in our study. LPS significantly induced the production of IL-6, TNF-α, eotaxin, and MCP-1 in the plasma (Figure 4). The increase in immobility duration in the TST and FST tests indicated the despair potency of the LPS-treated mice. These results are consistent with those of previous studies [31,41] (Figure 3). The LPS-treated mice were examined for inflammation along with depression after infection. Herbal medicines are typically administered for therapy after infection. Since baicalein is the main bioactive flavone of *Scutellaria baicalensis Georgi*, a traditional Chinese herbal medicine commonly used to treat inflammation, to test its therapeutic effect, baicalein was administered post LPS treatment (instead of pre-treatment) to mice in this study. The results demonstrate that baicalein administration significantly decreased the production of pro-inflammatory cytokines induced by LPS and normalized depression-like behaviors (Figure 3 and Figure 4).

Peripherally produced pro-inflammatory cytokines can access the brain through the choroid plexus and the circumventricular organs, stimulate afferent fibers of the vagus nerve, and then transmit inflammatory signals to the brain [42]. In another study examining inflammatory injuries in mice, peripheral TNF-α could activate microglia and induce the infiltration of monocytes into the brain [43]. Activated microglia and monocytes are thought to strengthen neuroinflammatory processes in the brain [44]. Our results demonstrated that baicalein had a remarkable ability to inhibit the production of pro-inflammatory cytokines in plasma, including that of TNF-α (Figure 4); therefore, it is reasonable to speculate that baicalein might indirectly modulate neuroinflammation. 

Accumulating evidence suggests that the involvement of neuroinflammation in affective disorders is complicated and profound [45]. In this study, neuroinflammation was evaluated by measuring the protein levels of NF-κB-p65 and iNOS in the hippocampus. Consistent with a recent report demonstrating that the levels of NF-κB and pro-inflammatory cytokines (IL-1β and TNF-α) significantly increased in the hippocampus of LPS-treated mice [46], LPS induced neuroinflammation and the production of NF-κB-p65 and iNOS in the present study. It is not surprising that the administration of baicalein significantly reduced the production of both proteins (Figure 6). NF-κB initiates the production and regulates inflammatory mediators, including iNOS, during neuroinflammation [47]. The reduction in the levels of iNOS prevents excessive NO production, which is cytotoxic to neurons and suppresses further inflammatory responses. Similarly, Du et al. demonstrated that baicalein treatment alleviated neuroinflammation and depression-like behavior in mice with experimentally induced autoimmune prostatitis (EAP) by inhibiting the NF-κB signaling pathway [48].

Downregulation of BDNF is associated with neuroinflammation in many brain disorders [20], which typically present with depression as a comorbidity. The relationship between BDNF and behavioral disorders has been demonstrated in many studies [49]. In this study, two types of active BDNF isoforms were examined: mature BDNF (mBDNF) and proBDNF (Figure 7A), which elicit contrary effects by binding to their receptors. Briefly, mBDNF signaling leads to neuronal survival and synaptic plasticity, whereas proBDNF signaling enhances neuronal death and synaptic pruning [50]. In rodents, the balance between mature BDNF and proBDNF has been proven to play an important role in depression-like behaviors induced by chronic unpredicted mild stress, and a decrease in the ratio of mBDNF/proBDNF could induce a reduction in the dendritic spine density of hippocampal neurons [51]. A strong relationship between BDNF, neural plasticity, and behavioral disorders has been demonstrated [52]. According to our results, LPS-induced production of mBDNF decreased significantly upon treatment with baicalein, and it seems that the production of proBDNF was not affected by LPS and baicalein. Baicalein reversed the LPS-induced reduction in the mBDNF/proBDNF ratio (Figure 7A–D). Preventing the downregulation of mBDNF might lead to neuronal survival, and reversing the ratio of mBDNF/proBDNF might be beneficial in building synapses. Baicalein is likely to protect neurons in these ways and exert an antidepressant response.

To further understand BDNF signaling, the protein levels of CREB in the hippocampus were examined. When mBDNF binds to its preferential receptor, TrkB, it activates several signaling cascades, such as the mitogen-activated protein kinase (MAPK) pathway and the phosphatidylinositol 3-kinase (PI3K) pathway, and eventually, CREB is activated [53]. CREB promotes the expression of the *BDNF* gene. We found that baicalein could elevate the protein levels of CREB in LPS-stimulated mice (Figure 7E,F), which might partially explain how baicalein inhibits the decrease in the production of mBDNF. 

As a reactive oxygen species (ROS) molecule, H_2_O_2_ may damage various biological processes via free radical oxidation. This study demonstrated that baicalein not only exhibited antioxidant activity in vitro (Figure 1) but also augmented the ability of mice to scavenge hydrogen peroxide in vivo (Figure 5), thereby protecting against LPS-induced oxidative stress. Gunawardena et al. had previously demonstrated that hydrogen peroxide mediates pro-inflammatory cell-to-cell signaling [54]. The strengthening of scavenging activity for H_2_O_2_ by baicalein is in line with the anti-inflammatory effects mentioned above.

Interestingly, we also observed that baicalein could mediate a reduction in the levels of IL-10 (Figure 6F), which is usually categorized as an anti-inflammatory cytokine. This indicates that the anti-inflammatory effect of baicalein cannot be attributed to IL-10 activity. In contrast, because baicalein attenuated inflammatory responses, the need for IL-10 to maintain the balance of the immune response was reduced. Macrophages are members of the innate immune system and play a vital role in mediating the inflammatory response. LPS, a toxin and an activator of macrophages, demonstrates a remarkable reduction in macrophage viability. We also found that baicalein could increase the viability of macrophages in vitro (Figure 2), which plays a critical role in the initiation, maintenance, and resolution of inflammation. Baicalein thereby protected macrophages from endotoxin-induced damage.

However, depression is highly prevalent, and its pathogenesis is complicated. Many patients do not have an optimal response to first-line antidepressant drugs, which are primarily designed to increase the availability of monoamines. The findings of this study demonstrate that baicalein might be a potential therapeutic agent for depression owing to its antioxidant and anti-inflammatory properties.

Although we demonstrated the antidepressant and anti-inflammatory effects of baicalein, this study still has some limitations. Based on the 3Rs principle (replacement, reduction and refinement), the number of mice in each group was 6–8. This small number might have caused bias. Additionally, to rule out the influence of the estrus cycle, only males were used; this is also a limitation in this study as the results are not generalizable for both sexes.

## 5. Conclusions

**Figure 8:**
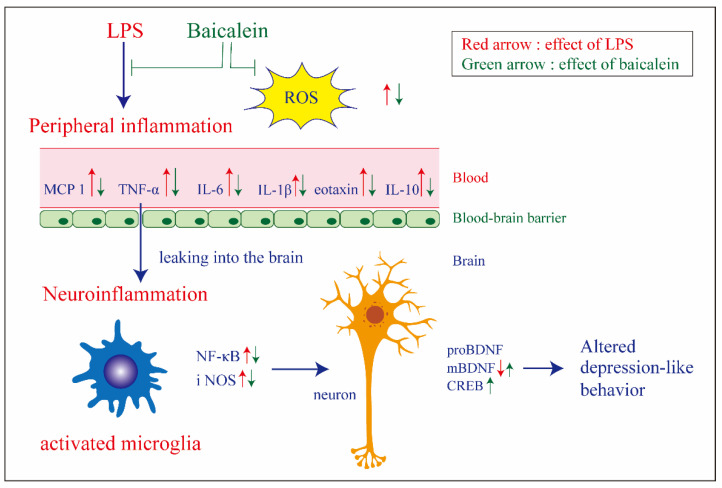
Graphic summary of the antidepressant effect of baicalein on LPS-induced depression-like behavior in mice and the possible mechanism involved. By decreasing the overproduction of reactive oxidative species (ROS) and pro-inflammatory cytokines (IL-6, TNF-α, MCP-1, and eotaxin), baicalein could attenuate neuroinflammation and restore the protein level of the mature brain-derived neurotrophic factor (mBDNF) in the hippocampus, thus contributing to the diminishing of depression-like behavior.

Taken together, baicalein attenuated LPS-induced depression-like behavior, and the possible underlying mechanism might involve its anti-inflammatory effects, including the reduction of plasma pro-inflammatory cytokines and iNOS and NF-κB protein levels in the hippocampus (Figure 8). BDNF and CREB play prominent roles in mediating the therapeutic antidepressant effect of baicalein; however, elucidation of detailed signaling mechanisms requires further research.

## Data Availability

The data presented in this study are available on request from the corresponding author. The data are not publicly available due to its proprietary nature.
